# Proteomics Research in Schizophrenia

**DOI:** 10.3389/fncel.2016.00018

**Published:** 2016-02-16

**Authors:** Katarina Davalieva, Ivana Maleva Kostovska, Andrew J. Dwork

**Affiliations:** ^1^Research Centre for Genetic Engineering and Biotechnology “Georgi D Efremov,” Macedonian Academy of Sciences and ArtsSkopje, Republic of Macedonia; ^2^Department of Molecular Imaging and Neuropathology, New York State Psychiatric InstituteNew York, NY, USA; ^3^Departments of Psychiatry and Pathology and Cell Biology, College of Physicians and Surgeons of Columbia UniversityNew York, NY, USA; ^4^Macedonian Academy of Sciences and ArtsSkopje, Republic of Macedonia

**Keywords:** brain tissue, 2D-DIGE, shotgun proteomics, glia, oligodendrocytes, astrocytes, myelin

## Abstract

Despite intense scientific efforts, the neuropathology and pathophysiology of schizophrenia are poorly understood. Proteomic studies, by testing large numbers of proteins for associations with disease, may contribute to the understanding of the molecular mechanisms of schizophrenia. They may also indicate the types and locations of cells most likely to harbor pathological alterations. Investigations using proteomic approaches have already provided much information on quantitative and qualitative protein patterns in postmortem brain tissue, peripheral tissues and body fluids. Different proteomic technologies such as 2-D PAGE, 2-D DIGE, SELDI-TOF, shotgun proteomics with label-based (ICAT), and label-free (MS^E^) quantification have been applied to the study of schizophrenia for the past 15 years. This review summarizes the results, mostly from brain but also from other tissues and bodily fluids, of proteomics studies in schizophrenia. Emphasis is given to proteomics platforms, varying sources of material, proposed candidate biomarkers emerging from comparative proteomics studies, and the specificity of the putative markers in terms of other mental illnesses. We also compare proteins altered in schizophrenia with reports of protein or mRNA sequences that are relatively enriched in specific cell types. While proteomic studies of schizophrenia find abnormalities in the expression of many proteins that are not cell type-specific, there appears to be a disproportionate representation of proteins whose synthesis and localization are highly enriched in one or more brain cell type compared with other types of brain cells. Two of the three proteins most commonly altered in schizophrenia are aldolase C and glial fibrillary acidic protein, astrocytic proteins with entirely different functions, but the studies are approximately evenly divided with regard to the direction of the differences and the concordance or discordance between the two proteins. Alterations of common myelin-associated proteins were also frequently observed, and in four studies that identified alterations in at least two, all differences were downwards in schizophrenia, consistent with earlier studies examining RNA or targeting myelin-associated proteins.

## The need for proteomics studies and biomarkers for schizophrenia

Genomic studies of schizophrenia, using genome wide association studies (GWAS), copy number variations (CNVs), microarrays, and next-generation sequencing (RNAseq) have linked schizophrenia with rare genetic variations (Sullivan et al., 2012). There is strong evidence that there are no known Mendelian variants identified for this disease. Instead, variations of many genes with confirmed involvement of rare structural variations and common variations with subtle effects are considered to be involved in the etiology of the disease (Owen et al., 2010). Genomics studies on schizophrenia have not answered the main questions on the pathophysiology of the disease, nor have they resulted in identification of diagnostic, prognostic, or therapeutic biomarkers. In addition, current research suggests that schizophrenia can arise from an interaction between neurodevelopmental processes and environmental effects (Albus, 2012).

Understanding schizophrenia as a complex disease therefore requires determination of not only gene expression and DNA variations, but also determination of the abundance and modifications of various proteins, and their distribution at gross anatomical, cellular, and subcellular levels. Proteomics aims to unravel biological processes based on qualitative and quantitative comparison of proteomes. It gives a different level of understanding than genomics for several reasons. First, the expression or function of proteins is modulated at many diverse points, from transcription of DNA to post-translational modifications (PTMs), and very little of this can be predicted from analysis of nucleic acids alone. Second, there is generally poor correlation between abundance of mRNA, transcribed from DNA, and abundance of protein translated from that mRNA. Third, many transcripts give rise to more than one protein, through alternative splicing or alternative PTMs such as phosphorylation, glycosylation, and acetylation, which profoundly affect their activities and lead to multiple protein products from the same gene.

Proteomic investigations have largely improved our understanding of schizophrenia, based on quantitative and qualitative identification of protein patterns in postmortem brain tissue, peripheral tissues, and body fluids (Martins-de-Souza et al., 2012b; Guest et al., 2013, 2014; Nascimento and Martins-de-Souza, 2015). This has enhanced our knowledge of complex protein networks and signal transduction pathways affected in this disease, as discussed in detail below. In addition, emerging proteomic platforms have facilitated the identification of biomarker candidates by simultaneous measurement of hundreds or thousands of molecules in non-hypothesis-driven comparative proteomics studies. This approach established the first blood-based test to aid in the diagnosis of schizophrenia (Schwarz et al., 2010). The test encompassed 51 biomarkers with an overall sensitivity and specificity of 83%. The clinical utility of this test has been studied in terms of specificity compared with other psychiatric disorders (Schwarz et al., 2012a), the ability to identify the disease prior to clinical manifestation, (Schwarz et al., 2012b) and the ability to define the complex schizophrenia syndrome on the basis of molecular profiles (Schwarz et al., 2014).

However, there is still an urgent need for biomarkers that will help to improve the diagnosis and stratification of patients and facilitate more effective treatments and care. The field of proteomics is rapidly developing, with improvements in mass spectrometry, peptide identification algorithms, and bioinformatics. Larger studies, standardized sample collection and processing, and highly sophisticated proteomics platforms promise new diagnostic, prognostic, and therapeutic biomarkers for schizophrenia.

## Proteomics studies of schizophrenia

The history of proteomic studies of schizophrenia largely recapitulates that of proteomics in general, driven over the past few decades by advances in the instrumentation and analysis of data for mass spectroscopy. We refer the reader to a recent publication (Doerr et al., 2015), in which these developments are reviewed for a general scientific audience.

The earliest proteomic investigations of schizophrenia employed two-dimensional polyacrylamide gel electrophoresis (2-D PAGE), combined with mass spectrometry (MS) identification, mainly based on matrix-assisted laser desorption/ionization-(MALDI)-time-of-flight (TOF)-TOF instruments. Moreover, 2-D PAGE/MS and later on, 2-D difference in-gel electrophoresis (DIGE)/MS are the most reported platforms in the study of postmortem brain tissues. As summarized in Table 1, 14 out of 25 proteomic studies on autopsy brain tissue from people with schizophrenia used 2-D PAGE/MS or 2-D DIGE/MS alone, and 2 studies employed combinations of these platforms with shotgun proteomics.

**Table 1:** **Proteins with altered abundance in schizophrenia obtained by comparative proteomics studies of several human brain regions**.

**Number of proteins with altered abundances**	Brain region (BA)^**^	Some of the proteins with altered abundances in schizophrenia compared to controls^*^	**Processes and pathways affected**	**Study design**	**Method**	**References**
**FRONTAL CORTEX**						
8 proteins	10	**DPYSL2**↓ **ALDOC**↑ **GFAP**↓	Differentiation, neurogenesis, glycolysis, development and organization of the central nervous system	Individuals with schizophrenia (*n* = 24), bipolar disorder (*n* = 23), major depressive disorder without psychotic features (*n* = 19) and unaffected controls (*n* = 23)	2-D PAGE/MS	Johnston-Wilson et al., 2000
**PREFRONTAL CORTEX**						
50 proteins	9 white and gray matter	**PKM**1↓; **PKM** 2↓; **ACO2**↓; **HK1**↓; **PRDX**1↓; **PRDX2**↓; **ALDOC**↓; **ALDOA**↓; **HSPA1**↓;**HSPA**2↓; **HSPA5**↓; **HSPA8**↓; **TUBA2**↓; **TUBA6**↓; **TUBB5**↓; **ENO1**/**2**↓; **GSN**↓; **GLUL**↓**; ACTA/B/C**↓; **MDH1**↓; **SPTAN1**↓; **CNP**↓**; DNM1**/2↓; **DPYSL2**↓; **DPYSL5**↓; **DPYSL**4↓; **SEPT**3↓; **TF**↑**; GSTM3**↓**, GSTTLp28**↓**;**	Mitochondrial function, oxidative stress responses, peroxisomal function, cytoskeletal dysfunction, protein trafficking	10 schizophrenia and 10 well-matched control individuals	2-D DIGE/MS	Prabakaran et al., 2004
15 proteins	9 (dorsolateral)	**DPYSL3**↑**;** PHGDH↑; **SEPT**5↑; **TCP1**↑; PGLS↑; **DNM1**↑; ERP29↑; ALB↑; **ENO2**↓; **GFAP**↑; GDA↓; **NEFL**↓; PACSIN1↓; **TF**↓; **ATP6V1B2**↓	Development of the central nervous system, energy metabolism, cytoskeletal dysfunction	35 individuals with schizophrenia, 35 with bipolar disorder and 35 controls	2-D PAGE/MS	Pennington et al., 2008
16 proteins	9 (dorsolateral) gray matter	LAMP↑; STXBP1↑; BASP1↑; NCAM↑; PHB↓; CNTN1↓; **CKB**↑; HBB↓; GNAI1↑; **TUBB/A**↑**; ATP6V1B2**↑; **VDAC1**↓	Synaptic function, metabolism, signaling, cytoskeletal dysfunction	Pooled samples from schizophrenia, bipolar disorder and control cases (*n* = 10 per group)	2-D DIGE/MS 1-D/LC-MS/MS	Behan et al., 2009
Targeted one protein expression	9 (dorsolateral)	**APOA1**↓	May have role in neuronal and glial metabolism	16 schizophrenia and 16 well-matched controls	Western blot	Huang et al., 2008
24 proteins	46 (left dorsolateral)	**MBP**↓; **TF**↓; **ALDOC**↑; **PGK1**↑; **ACO2**↑; **MDH1**↓; **ATP5H**↑; **ATP5A1**↑; UQCRC1↓; HSP90AA1 ↓; **HSPD1**↑; **INA**↑; **PCMT1**↑; **GFAP**↑; **NEFL**↓; **DNM1**↑; **CFL1**↓; **CRYM**↓; **GNB1**↑; **DPYSL2**↑**;ACTG1**↓	Alterations in oligodendrocytes, energy metabolism, glycolysis, oxidative stress, cytoskeletal dysfunction, cell signaling	pool of 9 schizophrenia patients and pool of 7 paired controls	2-D PAGE/MS	Martins-de-Souza et al., 2009a
84 proteins	46 (dorsolateral)	SIRPA↓; SIRPB1↓; **PRDX2**↑; **PRDX6**↑; PLEC↑; **VIM**↑; LMNB2↑; DES↑; **TUBB2B**↑; TBCB↑; KIF21A↑; **MAP1A**↑; MAP2↑; MAP6↑; EPB41L1↑; SH3GL2↑; **PEBP1**↑; BSN↑; HOMER1↑; STRN↑; **MOG**↓; CSNK2A1↑; MYO1D↓; **HK1**↑; PFKM↑; **GAPDH**↑; **CNP**↓	Immune system, cytoskeleton assembly, calcium homeostasis, energy metabolism, glycolysis	9 schizophrenia patients and 7 controls	ICPL /IEF/LC-MALDI	Martins-de-Souza et al., 2009c
32 proteins	46 (dorsolateral-white matter)	**NEFL**↓; **NEFM**↑; **NEFH**↓; **VIM**↑; **TUBB**↓**; YWHAE**↑; **YWHAZ**↓; **DPYSL2**↑**; DPYSL3**↑**; DPYSL5**↑**; PCMT1**↑; **STMN1**↓; GDI1↑; **ENO1**↓; **ALDOC**↓**; IMPA1**↑; **TPI1**↓; PRDX4↓; PDE8B↓,PLP1↑	Cytoskeletal dysfunction, signal transduction, energy metabolism	35 schizophrenia, 35 bipolar disorder and 35 controls	2-D DIGE/MS	English et al., 2009
41 protein spots	NS (left dorsolateral synapse enriched fractions)	**PKM**↓; **ATP6V1B2**↑; **ATP5B**↑; GDF2↓; GNAQ↑; **PEBP1**↑; ANXA10↑; **CKB**↑; GNB3↑; **PPA2**↑; **ALDOC**↑; AK4↑; **ANXA2**↓; HDGF↑; MLF2↑; **ATP5H**↑; **VDAC1**↑; PDYN↑;	Energy metabolism, neurotransmission/signaling	8 schizophrenia patients and 7 controls	2-D PAGE/MS	Smalla et al., 2008
Targeted one protein expression	46 (dorsolateral)	**HINT1**↓	May have role in cellular stress and apoptosis	9 schizophrenia patients and 7 controls	Western blot	Varadarajulu et al., 2012
Biomarker panel of 10 peaks	NS (dorsolateral)	No protein identifications (sensitivity and specificity of the panel 70%)		34 schizophrenia patients and 35 matched controls	SELDI-TOF-MS	Mei et al., 2006
Biomarker panel of 21 protein peaks	NS (dorsolateral)	CD58 ↓; CALM1↓; MAGOH↓; MAXb↓; RGS11↓; UCHL1↓; CEBPZ↓; **HSPD1**↓; **PGAM1**↓; **ENO2**↓**; GLUL**↓; **PPA2**↓; **YWHAE**↓; **ALDOC**↓; PNP↓; NKX2-4↓; DECR2↓; MTAP↓; BYSL↓; SMPD1↓; ANKRD12↓;	Cell metabolism, glycolysis, intracellular signaling, oxidative stress	17 schizophrenia patients, 20 bipolar disorder patients and 20 normal controls	SELDI-TOF-MS	Novikova et al., 2006
**ANTERIOR CINGULATE CORTEX**						
36 proteins	24 (gray matter)	**DPYSL2**↑; **CRMP1**↑; **ALDOC**↓**; DNM1**↑**; GFAP**↓**;** PPP1CA↓; **CS**↓; CA1↑; **TF**↓; IDH3B↓; ACAT2↑; **SOD1**↑; DDAH1↓; ANXA1↑; **ANXA5**↑; **CKB**↑**;** EHD3↑; **STMN1**↑; PHB↑;	Energy metabolism, oxidative stress, cytoskeletal dysfunction, protein trafficking, development and organization of the central nervous system	10 schizophrenia patients and 10 healthy controls	2-D PAGE/MS	Clark et al., 2006
27 proteins	24 (white matter)	**DPYSL2**↓; **ALDOC**↓**; CS**↑**;** CBR1↓; **ATP5A1**↑; **IMPA1**↑; GPD1↓; LDHA↓; **NEFL**↓; **ATP6V1A**↑; PSMB4↑; **PGAM1**↓	Energy metabolism, oxidative stress response, cytoskeletal dysfunction, cellular signaling	10 schizophrenia patients and 10 healthy controls	2-D PAGE/MS	Clark et al., 2007
19 proteins	NS	**ACO2**↑; **ALDOA**↑; **CRMP1**↑; **DPYSL2**↑; SRI↓; **CKB**↓;	Energy metabolism, glycolysis, cytoskeletal dysfunction	15 schizophrenia patients, 15 bipolar, 15 major depressive disorder and 15 healthy controls	2-D PAGE/MS	Beasley et al., 2006
32 proteins	NS	**DPYSL2**↑; **CRYAB**↑; **PRDX6**↓; **ALDOC**↑; **GLUL**↑	Energy metabolism, cell communication/ signal transduction, cytoskeletal dysfunction	11 schizophrenia patients and 8 healthy controls	2-D PAGE/MS	Martins-de-Souza et al., 2010b
143 proteins	24 (enrichment of the postsynaptic density)	ARF6↑; ARFGAP1↑; AP2B1↑; RAB11FIP5↑; AP2M1↓; ADRBK1↓; **DNM1**↑; **HSPA6**↓; **HSPA8**↓; RAP1A↑; GRIA2↓; MAPK3↓; SHANK3↓; SYNPO↓; MYL6↓; CYFIP2↓; ATP6V0A1↓; **PRDX1**↓; ESYT↑; GNAS↓; CAMK2B, CAMK2D↓; CAMK2G↓; CALM2↓; **VDAC1**↓; VDAC2↓	Endocytosis, long-term potentiation, calcium signaling pathway	10 schizophrenia patients and 10 healthy controls	Label-free LC−MS/MS	Föcking et al., 2015
**CORPUS CALLOSUM**						
34 proteins	NS	**NEFL**↓; **NEFM**↓; **GFAP**↑; **ACTG1** ↓; **ACTB** ↓; **TUBB**↓; **INA**↑; ACTR1A↑; COTL1↑; **ATP5B**↓; ARHGDIA↑; **STMN1**↑; **DPYSL2**↓; **YWHAH**↓; **HSPA8**↓; HSPA1L↓; **HSPA5**↑; **GSTP1**↑; **SOD1**↑; **CKB**↓**; PRDX2**↓; UCHL1↑; PARK7↑; FTH1↓; **LDHB**↓; **ENO2**↓**;**	Cytoskeletal dysfunction, oxidative stress, glycolysis, neuroprotective dysfunction	10 schizophrenia patients and 10 healthy controls	2-D PAGE/MS	Sivagnanasundaram et al., 2007
65 proteins	NS	**INA**↓**;** CLTC↓**;** VCAN↓**; FSCN1**↑**; GSN**↓**; NEFL**↓; HAPLN2↓**; NEFM**↓; **NEFH**↑; **TUBA1A**↓; **TUBA4A**↓; **TUBB2B**↓; **TUBB4B**↑; **TUBB3**↑; **TUBB6**↑;**STMN1**↑; **VIM**↑; **CNP**↓; **ENO1/2**↑; **ATP5A1**↓; **ALDOA**↓; GLUD1↑; **GAPDH**↓; PYGB↓; PYGM↓; CRYL1↓; **MDH1**↓; **PRDX1**↓; **PKM**↑; **SOD1**↓; **TPI1**↓; **HINT1**↑; **MOG**↓; **YWHAB/H/Z/G**↓; **CRYAB**↓; **HSPA6**↑; **UBE2N**↑; **MBP**↓; **ATP6V1B2**↑;	Cell growth and maintenance, energy metabolism, cell communication and signaling, protein metabolism, transport and myelination	9 schizophrenia patients and 7 healthy controls	Label-free nano LC-MS/MS	Saia-Cereda et al., 2015
Targeted one protein expression	NS	S100B↓	Disturbed oligodendrocyte maturation	9 schizophrenia patients and 7 healthy controls	2-D nano LC/MS Western blot	Steiner et al., 2014
**TEMPORAL LOBE**						
25 proteins	22 (left posterior superior- Wernicke's area)	**CKB**↑; **ALDOC**↓; **TPI1**↑; **GAPDH**↓; **PGAM1**↑; **ENO2**↑; **ATP6V1A**↑; **ATP5A1**↓; **QDPR**↑; **PRDX6**↑; **GFAP**↑; **PEBP1**↑**; ACO2**↑**; TUBB**↓	Energy metabolism, glycolysis	9 schizophrenia patients and 6 healthy controls	2-D PAGE/MS	Martins-de-Souza et al., 2009b
37 proteins	38 (left anterior)	**MOG**↓; **MBP**↓; **GFAP**↓; **UBE2N**↓; **CNP**↓; PPP3CA↓; **SPTAN1**↓, **YWHAH**↓; **YWHAZ**↓; **YWHAG**↓; **ALDOC**↓; HIST1H3A↓; CA2↓; RPS10↓; SLC9A3R1↑; **TUBB**↑; **NEFL**↓; **NEFM**↓	Oligodendrocyte-myelin regulated proteins, regulation Ca2+ homeostasis, energy metabolism	5 schizophrenia patients and 4 healthy controls	ICPL /IEF/LC-MALDI	Martins-de-Souza et al., 2009d
**HIPPOCAMPUS**						
34 proteins	anterior and posterior	Anterior **(CKB**↓; **ATP5H**↑; **LDHB**↓; NME1↑; COX5A↑; COX6B1↓; **PRDX2**↓; **PRDX6**↓; **SOD1**↓; **UBE2N**↑; PHB↑; HSPA1B↓; HSPA9↓; **ACTB**↓; **VIM**↑; **NEFM**↓; **STMN1**↑; FABP7↓; **YWHAZ**↑; **YWHAG**↑; **YWHAE**↑; **ANXA5**↑; **ATP6V1A**↓) Posterior **(PGAM1**↑**; CKB**↑**;** TALDO1↑**; GSN**↑**; ACTB**↑**; TUBA1**↓**; TUBB4**↓**; STMN1**↑**)**	Energy metabolism, oxidative stress, altered neurotransmission, mitochondrial dysfunction, abnormal neuronal and glial cytoarchitecture	Anterior hippocampus (7 schizophrenia patients and 7 controls) Posterior hippocampus (9 schizophrenia patients and 9 controls)	2-D PAGE/MS	Nesvaderani et al., 2009
108 proteins	mid	**GFAP**↓**; HSPA8**↓**;** CTSD↑; **STMN1**↑**; SOD1**↑**; DPYSL3**↓**; NEFL**↓**; FSCN1**↓; **ANXA6**↓**; TUB**↑**;** SEPT11↓; SNCA↓; **ATP6V1B2**↓**; ENO1**↓**; YWHAE**↓**;** PCMT1↓; **SPTAN1**↓; **PRDX1**↓;	cytoskeletal and metabolic changes, clathrin-mediated endocytosis	20 subjects with schizophrenia, 20 subjects with bipolar disorder, and 20 control cases	2-D DIGE/MS	Föcking et al., 2011
**MEDIODORSAL THALAMUS**						
10 proteins (2-D) 41 proteins (shotgun)	NS	TKT↑; **GSTP1**↑; **PGAM1**↑; **GAPDH**↑; **QDPR**↑; **TPI1**↑; **YWHAZ**↓; **CFL1**↑; **MBP**↑; **MOG**↑; **NEFM**↓; PRKCG↑; **GFAP**↓; **HSPA8**↓**; VIM**↑**; HSPA1A**↑**; ACT**↑**; GLUL**↑**; MAP1A**↑**;** MAPT↓; SYT5↑**;** SYN3↑; SEPT2↓	Energy metabolism, oligodendrocyte metabolism, cytoskeleton assembly	Samples from 11 schizophrenia patients and 8 controls	2-D PAGE/MS ICPL/IEF/LC-MALDI	Martins-de-Souza et al., 2010a
Targeted one protein expression	NS	**HINT1**↑	May have role in cellular stress and apoptosis	11 schizophrenia patients and 8 controls	Western blot	Varadarajulu et al., 2012

* *Proteins found in more than one independent study are highlighted in bold. The arrows indicate the increase (↑) or decrease (↓) of the protein level in the schizophrenia group compared to control group(s)*.

** *Brodmann area. NS, Brodmann area not specified or not applicable*.

Since its introduction in 1975 (O'Farrell, 1975), 2-D PAGE, combined with MS, has been widely used in proteomics studies. The platform is based on several steps of protein separation, detection, quantification and identification. Proteins are separated in two steps: by isoelectric point (pl) through isoelectric focusing on immobilized pH gradient (IPG) strips, followed by separation by molecular weight (MW) using SDS–PAGE. Proteins are detected using different staining protocols, and differences in abundances are quantified by image analysis software. The protein identification is based on excision of the 2D-spots of interest, enzymatic digestion (usually with trypsin), and analysis of the masses of these peptides by mass spectrometry [MALDI-TOF-MS or liquid chromatography (LC)–MS/MS]. Each protein produces a specific combination of peptide masses, known as a peptide mass fingerprint, which allows identification by comparison with database of fingerprints derived from protein sequences. To minimize ambiguity from gel-to-gel differences in 2-D PAGE, DIGE was introduced in 1997 (Unlü et al., 1997). DIGE combines multiple samples and internal standards in one gel by the fluorescent labeling of different samples with different cyanine dyes. This eliminates the need for technical replicates heavily used in conventional 2-D PAGE and improves reproducibility and sensitivity.

The advantages and limitations of this platform are well-established (Rabilloud and Lelong, 2011). The key advantages are high reproducibility, robustness, separation of intact proteins, visualization, and detection of PTMs, and cost-effectiveness of the procedure. A major disadvantage of any gel-based system, as recognized throughout the years, is limitation in the analysis of hydrophobic (membrane) proteins, high molecular weight proteins (Mw > 100 kDa), and highly acidic (pI < 3), or basic (pI > 9) proteins. Another important drawback is the limitation of the dynamic range of detection, as highly abundant proteins typically mask the identification of less abundant proteins with similar pI and MW. However, efforts to overcome these limitations are improving resolution (Butt and Coorssen, 2005) and increasing dynamic range (Wright et al., 2014a,b).

“Shotgun” proteomic techniques do not employ gels and are aimed to touch all possible targets at once. These are powerful tools for studying large-scale protein expression and characterization of complex biological systems. Shotgun methods begin with digestion of the whole proteome of interest, followed by high resolution separation of the resultant peptides by liquid chromatography (LC) and their identification based on tandem mass spectrometry (MS/MS) data; (Link et al., 1999). Increased resolution can be achieved by including pre-fractionation steps prior to LC-MS/MS. Pre-fractionation methods can include other types of chromatography (e.g., affinity chromatography), initial separation by isoelectric focusing (IEF), or one dimensional sodium dodecyl sulfate PAGE (1-D SDS PAGE). In the case of IEF and 1-D SDS PAGE, gels are divided into a number of pieces, and each piece is subjected to digestion and subsequent LC-MS/MS. IEF pre-fractionation has been used in 3 published studies of schizophrenia, and 1-D SDS PAGE pre-fractionation in one (Behan et al., 2009; Martins-de-Souza et al., 2009c,d, 2010b). In addition, the number of protein identifications can be increased by using two-dimensional liquid chromatography (2-D nano-LC), which has been done on postmortem brain tissue (Steiner et al., 2014). In shotgun proteomics, protein identification relies strongly on computational resources that combine and interpret data generated by MS/MS (Haas et al., 2006). The quantification of proteins in a shotgun-MS comparative analysis can be based on labeled or unlabeled peptides. Labeling methods used in schizophrenia proteomics studies include stable isotope labeling methods, such as isobaric tags for relative and absolute concentration (iTRAQ) and isotope-coded protein labeling (ICPL). However, most labeling-based quantification approaches have potential limitations, such as increased time and complexity of sample preparation, requirement for higher sample concentration, high cost of the reagents, incomplete labeling, requirement for specific quantification software, and a limited number of samples (2–8) per analysis. Most of these limitations, especially limits on number of samples, are eliminated in label-free approaches (Patel et al., 2009). The quantification in label-free proteomics is based on the theoretical assumption that the chromatographic peak areas of peptides correlate with their concentrations. Label-free proteomics analysis can be data-dependent (DDA; Stahl et al., 1996) or data-independent (MS^E^; Li et al., 2009), based on the selection of the peptide peaks for identification. In schizophrenia studies, MS^E^ has been used in several studies of peripheral tissues and body fluids (reviewed below) and is preferred over DDA because of the higher percentage of protein identifications and higher coverage of the proteome (Geromanos et al., 2009). MS^E^ was used to identify a serum biomarker panel capable of distinguishing first-onset drug-naive schizophrenia subjects from healthy subjects. This final panel was validated by immunoassay and later adapted into the multiplex immunoassay platform (MAP) leading to the first blood-based laboratory test for schizophrenia (Schwarz et al., 2010).

The strengths of the shotgun approach are experimental simplicity, greater proteomic coverage than gel-based platforms, and accurate quantification. Its weaknesses involve technical reproducibility, limited dynamic range, and informatics challenges related to the enormous complexity of the generated peptide samples (Domon and Aebersold, 2006). In addition, this approach cannot identify proteins with multiple modifications because the connection between the peptides that are analyzed in the mass spectrometer and the protein(s) from which the peptides originate is lost during proteolysis.

Surface-enhanced laser desorption/ionization time-of-flight mass spectrometry (SELDI-TOF-MS) has also been applied in the study of schizophrenia. SELDI-TOF is a variant MALDI-TOF that makes use of chemically-modified surfaces to reduce the complexity of biological samples prior to separation in the mass analyzer. Its invention increased interest in using patterns from mass spectra to differentiate samples with and without disease (Wright, 2002). SELDI provided several important advantages, such as ability to analyze complex biological samples with minimal pre-processing, ease of handling, and high throughput. However, over time, its lack of definitive protein identification and low reproducibility, influenced mainly by sample processing (Baggerly et al., 2004), became evident, and it is now used only rarely. There are several SELDI-TOF-MS schizophrenia studies of brain tissue and body fluids that appeared a decade ago, with and without identification of the affected proteins (Huang et al., 2006, 2008; Mei et al., 2006; Novikova et al., 2006; Craddock et al., 2008).

While all the proteomics platforms discussed have their strengths and weaknesses, it is becoming more widely accepted that combinations of different proteomics methods can take advantage of each method to increase the number of identified and quantified proteins, to detect and characterize PTMs and to increase statistical significance of the results. So far, schizophrenia studies combining 2-D PAGE or 2-D DIGE with shotgun proteomics are limited to one study of brain tissue (Behan et al., 2009), one study of both brain tissue and cerebrospinal fluid (Martins-de-Souza et al., 2010a) and one study of post-mortem pituitary glands (Krishnamurthy et al., 2013). However, as the growing number of studies demonstrate the power of combining these two platforms, it seems that a combination of gel-based and shotgun proteomics will be the best route to an understanding of the biological pathways of schizophrenia and to the discovery of reliable biomarkers for diagnosis, stratification and therapy.

Combinations of proteomics techniques other than gel-based and shotgun proteomics reportedly result in high confidence biomarker discovery. MALDI mass spectrometric imaging (MSI), combined with high resolution top-down tandem MS identification, has been used for the discovery of biomarkers for neurodevelopment disorders (Ye et al., 2014) but has not yet been used to study schizophrenia. MALDI MSI is a relatively new imagining technique that is label-free and enables simultaneous mapping of proteins and numerous molecules in tissue samples with great sensitivity and chemical specificity (Seeley and Caprioli, 2008). It has enabled discovery of tissue biomarkers for various cancers. The top-down tandem MS approach differs from the shotgun approach by directly analyzing intact proteins and allowing assessment of protein modifications, such as PTMs and sequence variants, with no prior knowledge (Kelleher, 2004; Michalski et al., 2012). This combination is reported to be fast and robust, and it has provided highly reproducible proteome “snapshots” of anatomical regions in sections of infant rat brain. It has high potential for future studies in schizophrenia.

## Candidate proteins implicated in schizophrenia

### Tissue biomarkers

As schizophrenia is presumably a brain disease, the comparative proteomic analysis of human postmortem brain tissue is of highest interest as it may reveal the disease-related proteins. This may help in the understanding the molecular mechanisms of the disease and in addition indicate potential biomarkers as candidates for diagnosis, prognosis, and therapy.

We have found 25 published articles on the proteomics of human brain tissue in schizophrenia. These studies investigated the relationship between schizophrenia and the protein profiles of six distinct brain regions: frontal or prefrontal cortex, anterior cingulate cortex, corpus callosum, temporal lobe neocortex, hippocampus, and mediodorsal thalamus. Detailed review of these studies regarding the study design, brain regions, number of identified proteins with altered abundancies, some of the candidate biomarker proteins, affected pathways in relation to schizophrenia and proteomics platforms used, are given in Table 1.

The most extensively studied brain region by proteomics studies is prefrontal cortex (PFC). The primary functions of PFC include the organization of thoughts and actions (cognitive control) such as willed action, decision making, and working memory (Frith and Dolan, 1996; Miller and Cohen, 2001). Individuals with schizophrenia exhibit poor working memory (Park and Holzman, 1992), abnormal eye movements (Park and Holzman, 1993), and abnormal executive functioning (Velligan and Bow-Thomas, 1999). Well-known abnormalities of dorsolateral (DL) PFC reported in schizophrenia include failure to activate during the Wisconsin card-sorting test (Daniel et al., 1991), decreased expression of calcium-binding proteins and glutamic acid decarboxylase in interneurons (Hashimoto et al., 2003), decreased density of spines on pyramidal cell dendrites (Glantz and Lewis, 2000), and decreased cortical volume without loss of neurons (Selemon et al., 1995).

The first proteomics study of schizophrenia (Johnston-Wilson et al., 2000) used 2-D PAGE/MS to compare PFC, specifically Brodmann area (BA) 10, of *post mortem* human brains from individuals with schizophrenia, individuals with other psychiatric disorders, and “controls” (i.e., individuals who died without history of psychiatric disorder). Half of the published proteomics studies on schizophrenia so far have investigated the PFC, mostly dorsolateral PFC (Brodmann areas 9 and 46; Prabakaran et al., 2004; Mei et al., 2006; Novikova et al., 2006; Huang et al., 2008; Pennington et al., 2008; Smalla et al., 2008; Behan et al., 2009; English et al., 2009; Martins-de-Souza et al., 2009a,c; Varadarajulu et al., 2012). Different proteomics platforms were used, mostly gel-based platforms (2-D PAGE/2-D DIGE) followed by shotgun proteomics or SELDI-TOF-MS. Western blot was used for targeted analysis of particular proteins of interest or validation of selected candidates.

The anterior cingulate cortex (ACC) is involved in emotion and behavior (Luu and Posner, 2003). The association between ACC and schizophrenia was based on abnormal activation of the ACC during hallucinations (Cleghorn et al., 1990) and task performances (Carter et al., 1997; Quintana et al., 2004) and on histological abnormalities (Benes et al., 2001; Bouras et al., 2001; Chana et al., 2003; Salgado-Pineda et al., 2003). The differential protein expression between schizophrenia and non-psychiatric groups was investigated in 4 studies using 2-D PAGE/MS (Beasley et al., 2006; Clark et al., 2006, 2007; Martins-de-Souza et al., 2010b) and one recent study using LC-MS/MS to study expression of proteins in postsynaptic densities (Föcking et al., 2015).

Neuropathological and neuroimaging studies have repeatedly reported structural abnormalities of the corpus callosum (CC) in schizophrenia, such as smaller volume, poor structural integrity of the axonal fiber tracts, and decrease in density of axons (Shenton et al., 2001; Mehler and Warnke, 2002; Innocenti et al., 2003). CC has been investigated by two comparative proteomics studies using 2-D PAGE/MS (Sivagnanasundaram et al., 2007), and LC-MS (Saia-Cereda et al., 2015), respectively. There is one more proteomics study of corpus callosum (Steiner et al., 2014), which used targeted 2-D nano LC/MS and western blot, to analyze S100B protein. In temporal lobe (TL) neocortex, Wernicke's area (posterior region of Brodmann area 22) and left temporal pole (Brodmann area 38) have been implicated in the pathophysiology of schizophrenia because of their roles in speech, language, and communication. There are two comparative proteome analyses of these brain regions, performed by 2-D PAGE/MS and shotgun proteomics (Martins-de-Souza et al., 2009b,d).

Hippocampus and mediodorsal thalamus (MDT) are both areas of interest in schizophrenia where early studies reported structural abnormalities that were later called into question (Dwork, 1997; Romanski et al., 1997; Harrison and Eastwood, 2001) and were subsequently subjected to proteomics investigation. There are 2 comparative studies of hippocampus using gel-based proteomics (Nesvaderani et al., 2009; Föcking et al., 2011), 1 comparative study of MDT using combination of 2-D PAGE/MS and shotgun proteomics (Martins-de-Souza et al., 2010a), and one targeted analysis of specific protein expression in mediodorsal thalamus by Western blot (Varadarajulu et al., 2012).

What is common to all of the proteomics studies of brain tissue is that, regardless of the platform and brain region analyzed, there has been constantly observed alteration of energy metabolism expressed as disturbed levels of proteins included in glycolysis, Krebs cycle, mitochondrial function, oxidative stress, and various other energy pathways. Abnormalities of proteins mediating synaptic function and signal transduction were also observed.

The central pathway for energy metabolism is glycolysis. Proteome analyses of different brain regions from individuals with schizophrenia have consistently revealed a number of differentially expressed glycolytic enzymes, as shown in Table 1. Aldolase C and A (ALDOC/A), have been found with differential abundance in 13 proteomics studies investigating FC, PFC, ACC, CC, and TL. Alpha and gamma enolase (ENO1/2) were found with differential abundance in 8 studies of PFC, CC, TL, and hippocampus. Glyceraldehyde-3-phosphate dehydrogenase (GAPDH), triosephosphate isomerase (TPI1), and phosphoglycerate mutase 1 (PGAM1) were also found with differential abundance in all of the investigated brain regions except FC, while dysregulation of hexokinase (HK1) was observed in two studies of PFC. Dysregulation of lactate dehydrogenase complex (LDHA/B), which reduces pyruvate into lactate and represents the intersection of key pathways of energy metabolism, was observed in 3 studies analyzing ACC, CC, and hippocampus.

Dysregulation of the Krebs cycle has also been implicated in schizophrenia as a result of differential abundance of several involved enzymes: such as aconitase 2 (ACO2), malate dehydrogenase 1 (MDH1), and citrate synthase (CS). The dysregulation of these enzymes was mainly observed in the ACC, 2 studies of PFC, and 2 studies of CC and TL.

Dysregulation of different ATP synthase subunits, such as ATP5A1, ATP5B, and ATP5H in PFC, ACC, CC, TL, and hippocampus suggests abnormalities of mitochondrial function. Overall dysregulation of energy metabolism leads, among other consequences, to overproduction of reactive oxygen species (ROS) and oxidative stress. Oxidative stress events present in schizophrenia and have been identified on a proteomic level through dysregulation of several groups of enzymes, mainly peroxiredoxins (PRDXs), glutathione transferases (GST), NADPH-dependent oxidoreductases, and antioxidant enzymes. The most prominent dysregulation was observed at peroxiredoxins (PRDX1, 2, 3, 6) found with differential abundancies in 11 proteomics studies and in all investigated brain regions except MDT. Glutathione S-transferase P (GSTP1), carbonyl reductase 1 (CBR1), quinoid dihydropteridine reductase (QDPR), and antioxidant enzyme superoxide dismutase (SOD1) were also found with differential abundances in several studies and various brain regions, but not in FC and PFC. From the proteins involved in other energy pathways, vacuolar ATP synthases (ATP6V1A, ATP6V1B2, ATP6V0A1) and creatine kinase (CKB) were consistently found with differential abundance in all of the investigated brain regions of schizophrenia patients. The rest of the proteins from this group (glutamine synthase [GLUL], transketolase [TKT], and carbonic anhydrase 1 and 2 [CA1/2]) were found with differential abundance in some brain regions, as presented in Table 1.

As with the proteins involved in energy metabolism, proteomics studies of the brain tissue consistently revealed changes in the abundance of some of the cytoskeletal components of the cell. These encompassed 4 major components: (1) microfilaments composed of actin, (2) microtubules composed of tubulin, (3) intermediate filaments, which are a family of about 70 different proteins, and (4) microtubule associated proteins. Differential abundance of beta and gamma actin isoforms was observed in 8 studies, including three studies of dorsolateral PFC, while the others included all other investigated brain areas. Alpha and beta tubulins were also found with differential abundance in 12 independent studies, mainly in PFC, ACC, CC, and hippocampus. There were 5 intermediate filament proteins with disturbed abundance in schizophrenia: desmin (DES), vimentin (VIM), glial fibrillary acidic protein (GFAP), and neurofilament medium and light chains (NEFM and NEFL). The most prominent change inivolved neurofilament proteins, observed in 11 studies, followed by GAFP in 9 studies, and in all investigated brain regions. Disturbed levels of VIM and DES were observed less frequently, in 5 (English et al., 2009; Martins-de-Souza et al., 2009c, 2010b; Nesvaderani et al., 2009; Saia-Cereda et al., 2015) and 1 study (Martins-de-Souza et al., 2009c), respectively. Microtubule-associated proteins (MAPs) with differential abundance in schizophrenia encompassed microtubule-associated proteins 1A (MAP1A), 2 (MAP2), and tau (MAPT), and dynamin 1 (DNM1). The differential abundance of these proteins was observed at lower rates than were other cytoskeletal proteins. Changed levels of DNM1 were found in 5 studies of which 3 assayed PFC (Prabakaran et al., 2004; Pennington et al., 2008; Martins-de-Souza et al., 2009a) and 2 assayed ACC (Clark et al., 2006; Föcking et al., 2015), while MAP1A and MAPT were found in only 2 studies, 1 of PFC (also including MAP2) and 1 of MDT (Martins-de-Souza et al., 2009c, 2010b).

Cytoskeleton associated proteins dihydropyrimidinase-related protein 2 (DPYSL2), 14-3-3 protein family (YWHAE, YWHAZ, YWHAG, YWHAH, YWHAB), spectrin (SPTAN1), stathmin (STMN1), and various representatives from the heat shock proteins (HSPD1, HSPA5, HSPA8, HSPA1L, HSPA1A, HSPA1B) were detected with abnormal abundance in more than 5 independent studies, providing further support for cytoskeletal dysfunction in schizophrenia.

Oligodendrocyte dysfunction in schizophrenia has been suggested by brain imaging, epigenetic and transcriptomics studies, discussed in detail elsewhere (Dwork et al., 2007; Martins-de-Souza, 2010; Haroutunian et al., 2014). Six proteomics studies from 2 independent groups so far have found altered expression of some of the oligodendrocyte-related proteins in schizoprenia brain tissues such as 2′,3′-cyclic nucleotide 3′-phosphodiesterase (CNP), myelin basic protein (MBP), myelin oligodendrocyte glycoprotein (MOG), and gelsolin (GSN; Prabakaran et al., 2004; Martins-de-Souza et al., 2009a,c,d, 2010b; Saia-Cereda et al., 2015). We found 5 proteomics studies focused on white matter. Three (Sivagnanasundaram et al., 2007; Steiner et al., 2014; Saia-Cereda et al., 2015) examined corpus callosum. One (Steiner et al., 2014) was focused on S100B, reportedly elevated in blood and CSF of individuals with schizophrenia, and found a decrease of 50–70% by mass spectroscopy and western blot. The more recent (Saia-Cereda et al., 2015) of the other two studies, employing label-free nano LC/MS/MS, identified downregulation of CNP, MBP, MOG, and GSN, while they were not found to be abnormal in the older study (Sivagnanasundaram et al., 2007), which employed 2-D PAGE/MS. Proteins involved in filaments and microtubules were abundantly represented in the results from both studies, although fiber densities in the corpus callosum have mostly been found unaffected by schizophrenia (reviewed in Dwork et al., 2007). The other two proteomics studies analyzed post mortem white matter from PFC (English et al., 2009) and ACC (Clark et al., 2007). Both found reduced levels of ALDOC in schizophrenia. Among myelin-related proteins, the only change in schizophrenia was upregulation of PLP1 in prefrontal white matter. In contrast, *in situ* hybridization of ACC white matter showed downregulation of mRNA for CNP and MAG in schizophrenia (McCullumsmith et al., 2007). In a targeted study (Dracheva et al., 2006), western blot for CNP showed decreased levels in the hippocampus but not the putamen from individuals with schizophrenia.

One study (Krishnamurthy et al., 2013; included in Table 2) employed several platforms to study pituitary gland from autopsies of individuals with schizophrenia or without psychiatric illness. The pituitary glands from subjects with schizophrenia showed disturbance in hypothalamic- pituitary-adrenal axis, immune system, lipid transport, and metabolism. By LC-MS^E^, prolactin was significantly lower, by approximately 35% in pituitary from chronically medicated individuals with schizophrenia, suggesting that despite antipsychotic medication, prolactin production was suppressed by elevated dopamine. While the same average difference was found by western blot, for schizophrenia and bipolar disorder as well, the differences from normal were not significant. By contrast, western blot gave virtually identical levels for follicle stimulating hormone across all diagnostic groups.

**Table 2:** **Candidate protein biomarkers for schizophrenia obtained by proteomics studies using human body fluids and tissues other than brain**.

**Number of proteins with altered abundances**	**Body fluid**	**Candidate biomarkers**	**Processes and pathways affected**	**Study design**	**Method**	**References**
1	CSF	**APOA4**↓	Reverse cholesterol transport	10 controls and 10 schizophrenia patients were pooled to form four pools for each group	2-D PAGE/MS	Jiang et al., 2003
2	CSF	VGF 23-62 peptide↑; **TTR**↓	Synaptic plasticity, penile erection, circadian clock, thyroid hormone binding	58 schizophrenia, 16 with depression, 5 with obsessive-compulsive disorder, 10 with Alzheimer disease and 90 controls	SELDI-TOF-MS	Huang et al., 2006
1	CSF	**APOA1**↓	Plasma cholesterol esters formation	CSF: 41 vs. 40; liver:15 vs. 15; RBC: 20 vs. 20; serum: 35 vs. 63; brain 1st exp: 8 vs. 8; brain 2nd exp: 9 vs. 9	SELDI-TOF-MS	Huang et al., 2008
6	CSF	**APOE**↑; **APOA1**↑; **TTR**↓; TGFBR1↓; CCDC3↓; PTGDS↑	Molecular transport, signal transduction receptors	17 first episode schizophrenia and 10 controls	Western blot	Martins-De-Souza et al., 2010c
7	plasma	**HP**↑; **SERPINA1**↑; **CFB**↑; **APOA1**↓; **APOA4**↑; **TTR**↓	Acute phase proteins, molecular transport	22 schizophrenia patients and 20 controls	2-D PAGE/MS	Yang et al., 2006
6	plasma	**HP**↑; **SERPINA1**↑; **APCS**↑; **AMBP**↑; SERPINC1↑; GC↑	Acute phase, coagulation and transport	42 schizophrenia patients and 46 healthy controls	2-D PAGE/MS	Wan et al., 2007
1	plasma	α-defensins (**DEFA**) ↑	Dysregulation of immune pathway of peripheral white blood cells	T cell lysates from 15 schizophrenia and 15 controls	SELDI-TOF-MS	Craddock et al., 2008
79	plasma	BDNF↑; EGF↑; CXCL5↑; TIMP1↑; **MMP9**↑; **Insulin**↑	Synaptic transmission, growth factors, chemo-attractants, proteolytic system	245 major depressive disorder, 229 schizophrenia and 254 controls	Multiplexed immunoassay	Domenici et al., 2010
10	serum	**CD5L**↓; **IGHM**↓; F13B↓; TF↓; **AHSG**↓; APOD↓; **APOA1**↓; **APOA4**↓; **APOA2**↓; **APOC1**↓	Lipid metabolism, cholesterol transport pathway, immune response	22 first onset schizophrenia patients and 33 matched controls	Label-free nano LC-MS/MS (MS^E^)	Levin et al., 2010
181 tested	serum	51 biomarker panel	Diverse protein functions	577 schizophrenia patients and 229 controls	Multiplexed immunoassay	Schwarz et al., 2010
21 tested (7 selected)	serum	**Insulin** ↑; **CHGA**↑; PPY↑; PRL↑; Cortisol↑; GH1↓; Progesterone↑;**prolactin**↑ growth hormone↓	Dysregulation of glucose metabolism, hypothalamic-pituitary-adrenalgonadal axis hormones	236 first and recent onset schizophrenia patients and 230 matched controls	Multiplex immunoassay; 2D-DIGE	Guest et al., 2011
181 tested	serum	34 biomarker panel	Diverse protein functions	250 first and recent onset schizophrenia, 35 major depressive disorder, 32 bipolar disorder, 45 Asperger syndrome and 280 control subjects	Multiplexed immunoassay	Schwarz et al., 2012a
20	serum	SPP1↓; CALB1↑; CTGF↑; TBG↑; **APOA1**↑; APOB↑; **CHGA**↑; **AMBP**↓; IL6R↓; IL17↓; **CD5L**↑; **PROS1**↑; **APCS**↑; CA125↓; TFF3↓; GOT1↓; **HGF**↓	Inflammation and immune response	75 schizophrenia, 110 bipolar disorder patients and their matched controls (75+110)	Multiplexed immunoassay	Schwarz et al., 2012b
191 tested	serum	Symptom severity (FABP5; **CKB**; MB; CRP; MMP9; **HGF**) Response prediction (**insulin**) Time to relapse (LEP↓; **proinsulin** ↓; TGFA↑; **CD5L**↓; B2M↓; **MMP9**↑; CD40↓; **APOC1**↓)	Acute phase response pathway, transport, immune response, glucose metabolic pathways	77 schizophrenia patients were analyzed to identify molecules associated with symptom severity, predict response over a 6-week treatment period and predict the time to relapse	Multiplexed immunoassay	Schwarz et al., 2012c
35 (additional 59 with only phosporilation pattern changes)	serum	Altered abundance and phosphorylation: ALS2↑; MAST1↑; **CFB**↑; C4BPA↑; CFHR3↑; C6↑; ITIH3↑; **APOA1**↓; MYOF↓; **APOA2**↓; CCDC57↓; SMC1A↓; GPLD1↓	Acute phase response signaling pathway, Complement system, LXR/RXR activation, Coagulation system, Intristic prothrombin activation pathway	20 antipsychotic-naïve schizophrenia patients and 20 matched healthy controls	IMAC/LC-MS/MS (MS^E^)	Jaros et al., 2012
27	serum	ANPEP↓; APOC2↓;APOF↓; APOL1↓; C4BPB↓; C8B↑; **CD5L**↑; DBH↑; F7↓; GGH↓; ICAM2↓; IGFALS↓; IGHG4↑; SERPINA5↑; **IGHM**↑; ITIH4↑; KNG1↑; LBP↓; PGLYRP2↑; PI16↑; PLTP↑; **PROS1**↓; ZNF57↓	Complement system, coagulation cascades	10 antipsychotic drug- naïve schizophrenia patients and 10 healthy volunteers	LC-MS/MS	Li et al., 2012
23 immune molecules and 30 growth factors and hormones	serum	group 1 (MIF, CXCL8, IL1RN, IL18, and IL16) group 2 (**prolactin**, resistin, testosterone, **insulin**, PDGFRA, LEPROT, and AGT)	Identified 2 subgroups of schizophrenia patients based on molecular serum profiles. Group 1 had changes in immune molecules, and group 2 changes in growth factors and hormones	180 antipsychotic-naive schizophrenia patients and 398 matched controls	Multiplexed immunoassay	Schwarz et al., 2014
5	blood	**Insulin**↑; **proinsulin**↑; des-31,32-proinsulin↑; C-peptide↑; **CHGA**↑	Dysregulation of glucose metabolic pathways	66 schizophrenia, 10 bipolar and 78 matched controls	Fluorescence assays and immunoassays	Guest et al., 2010
17 validated by MRM/MS	sweat	AZGP1↑; ANXA5↑; TXN↑; ARG2↑; BLMH↑; CDSN↑; CALML5↑; CASP14↑; **CSTA**↑; DSG↑; PARK7↑; G3PDH↑; KLK11↑; PSBP1↑; S100A7↑; **PRDX1**↑; PIP↓	Oxidative stress, lipid metabolism, Ca and others binding proteins, epidermal differentiation and integrity, proteinase inhibitors, cell-cell adhesion, glycolysis	The first set was made by pooling of 8 patients samples and 8 controls. The second set was made by pooling of 4 patients and 4 controls	LC−MS/MS; MRM/MS	Raiszadeh et al., 2012
8	saliva	**DEFA1-4**↑; S100A12↑; **CSTA**↑; S-derivatives of CSTB↑	Dysregulation of immune pathway of peripheral white blood cells	32 schizophrenia, 17 bipolar and 31 healthy controls	RP-HPLC–ESI-MS (top-down)	Iavarone et al., 2014
18	peripheral blood mononucl-ear cells (PBMCs)	Unstimulated PBMCs (CNDP2↑; COTL1↓; GPI↓; **HSPA2**↓; **LDHB**↑) Stimulated PBMCs (**ALDOC**↑; **HSPD1**↓; **GAPDH**↑; HNRPK↑; **LDHB**↑; NAMPT↑; MYH14/15↑; **PGK1**↑; PPIA↑; **TPI1**↑; PKLR↑; PGAM4↑)	Dysregulation of glucolytic pathway	12 first onset schizophrenia patients, 7 chronically ill antipsychotic treated schizophrenia patients and 19 healthy controls	Label-free nano LC-MS/MS (MS^E^)	Herberth et al., 2011
29 (LC-MS^E^) 13 (2-D DIGE)	pituitary gland	LC-MS^E^ (SIPA1↑; CEMIP↑; FGB↑; CFAP43↑; MYH9↑; HNRNPA2B↓TTC28↓; PIWIL3↓; **APOA1**↓; **APOA2**↓; **CKB**↓; **TUBA1A**↓; **TUBB1B**↓; **TUBB2C**↓; **AHSG**↓; TGM2↓; TUBB4B↓; LMX1B↓; CTNND2↓; YWHAQ↓; **HP**↓; **CRYM**↓; HSPB1↓; SCGN↓; SETD5↓; TIAM2↓;	Disturbance in hypothalamic—pituitary—adrenal axis, immune system, lipid transport and metabolism	14 schizophrenia and 15 control subjects were analyzed by comparative proteomics. Additional 13 bipolar disorder and 14 major depression patients were included in Western validation	LC-MS/MS (MS^E^) Multiplexed immunoassay, 2-D DIGE, Western blot	Krishnamurthy et al., 2013

*Candidate biomarkers found in more than one independent study are highlighted in bold. The arrows indicate the increase (↑) or decrease (↓) of the protein level in schizophrenia group compared to control group(s)*.

### Body fluids biomarkers

The comparative proteomics analysis of body fluids is of highest clinical interest as it may reveal biomarkers for diagnosis, choice of therapy and future course of schizophrenia. We found 20 proteomics studies investigating potential schizophrenia biomarkers in cerebrospinal fluid, blood, sweat, and saliva, and 2 additional studies investigating protein alterations in peripheral blood mononuclear cells (PBMCs) and the aforementioned study of pituitary gland (Table 2).

Cerebrospinal fluid (CSF) and blood are the main body fluids used in proteomic studies of neurological or psychiatric disorders, including schizophrenia. CSF is presumed to be representative of the extracellular space in the CNS. Consequently, pathological abnormalities of the brain might be directly reflected in CSF. Normal CSF protein concentrations are ~0.5% those of normal serum or plasma, requiring highly sensitive analytical techniques. Fewer proteins can normally be detected. CSF sampling is moderately invasive, so it is difficult to perform longitudinal studies. The first 2-D map of human CSF proteome was presented by Goldman and collaborators in 1980 (Goldman et al., 1980). With the development of proteomics technologies, different approaches were used for discovering CSF biomarkers for schizophrenia: 2-D PAGE and 2-D DIGE followed by MALDI-MS identification (Jiang et al., 2003; Martins-De-Souza et al., 2010c) and SELDI-MS (Huang et al., 2006, 2008). These studies revealed altered levels of proteins involved in molecular transport of cholesterol and of proteins involved in phospholipid metabolism [apolipoprotein A-I (APOA1), apolipoprotein A-IV (APOA4), and apolipoprotein E (APOE), 23-62 VGF peptide], transthyretin (TTR), arachidonic acid metabolism enzyme-prostaglandin-H2 D-isomerase (PTGDS), coiled-coil domain-containing protein 3 precursor (CCDC3), and transforming growth factor-β receptor type-1 (TGFBR1).

The major advantage of using blood for biomarker discovery arises from two main blood characteristics. With the exception of the urine, which usually contains no protein, blood is the most easily and least invasively sampled body fluid for clinical studies. This accessibility allows multiple samplings at various stages of the disease and treatment. Second, blood interacts with all tissues, and therefore can indicate changes anywhere in the body.

We found 14 proteomic studies investigating potential biomarkers in plasma and serum. The first 2 studies investigated the plasma proteome by 2-D PAGE coupled with MS. They revealed altered levels of acute phase response proteins (Yang et al., 2006; Wan et al., 2007), suggesting a link between inflammatory response systems and pathophysiology of schizophrenia. The affected proteins included haptoglobin (HP), α-1-antitrypsin (SERPINA1), complement factor B precursor (CFB), apolipoprotein A1 (APOA1), apolipoprotein A4 (APOA4), transthyretin (TTR), serum amyloid P-component (APCS), alpha-1-microglobulin (AMBP), and antithrombin III (SERPINC1). A study using a SELDI-TOF platform to analyze lysates of stimulated T lymphocytes found alterations of α-defensin (DEFA) in minimally medicated subjects with schizophrenia, and also in their monozygotic twins without schizophrenia (Craddock et al., 2008), suggesting a marker for susceptibility.

There are 3 studies of serum proteome so far using label-free LC-MS/MS. The first study of serum proteome found that the most significantly changed proteins belong to the apolipoprotein family (apolipoprotein D [APOD], apolipoprotein A1 [APOA1], apolipoprotein A4 [APOA4], apolipoprotein A2 [APOA2], and apolipoprotein C1 [APOC1]), involved in the metabolism of high-density lipoprotein and triglyceride-rich lipoproteins, and in the reverse cholesterol transport pathway. These were followed by proteins involved in immune response (CD5 molecule-like [CD5L], IgM [IGHM], transferrin [TF]) and in insulin resistance (α-2-HS glycoprotein [AHSG]; Levin et al., 2010). Subsequent studies (Jaros et al., 2012; Li et al., 2012) confirmed that subjects with schizophrenia feature serum abnormalities in levels and phosphorylation of proteins involved in immune response. These studies also implicated the complement and coagulation cascades as the most significant pathways disturbed in schizophrenia.

However, the majority of the studies on plasma and serum have used multiplexed immunoassays containing selected subsets of biomarkers, constructed based on the findings from previous discovery proteomics studies. The first attempt to develop a molecular test with clinical utility for diagnosis of schizophrenia resulted in formation of a 51-plex biomarker panel with sensitivity and specificity of 83% (Schwarz et al., 2010). The study of specificity of this panel for schizophrenia compared with other psychiatric disorders led to establishment of a reduced, 34-biomarker panel that gave a separation of 60–75% of schizophrenia subjects from controls across 5 independent cohorts (Schwarz et al., 2012a). In the most recent studies, the multiplexed immunoassay test was also investigated for the ability to identify the disease prior to clinical manifestation (Schwarz et al., 2012b) and to characterize subgroups of individuals with schizophrenia on the basis of the molecular pathways affected (Schwarz et al., 2014).

Several multiplex immunoassay studies identified elevated insulin-related peptides in untreated first-onset schizophrenia subjects, suggesting that dysregulation of glucose metabolic pathways may have a role in the development of schizophrenia (Guest et al., 2010, 2011; Schwarz et al., 2012c). Immune response alterations have also been observed by several studies (Schwarz et al., 2012b,c). Also, altered levels of hormones (prolactin, cortisol, progesterone, and growth hormone) in blood have been observed in people with schizophrenia, indicating that functions of multiple components of the hypothalamic-pituitary-adrenal-gonadal axis may be affected (Domenici et al., 2010; Guest et al., 2011).

There is only one report describing sweat proteome alteration in schizophrenia (Raiszadeh et al., 2012). The eccrine sweat glands, controlled by the sympathetic nervous system, are responsible for thermoregulation. Distributed over the entire body, they produce a fluid that is composed of inorganic ions, lactate, urea, ammonia, amino acids, and proteins, including plasma and epidermal proteins. The study of Raiszadeh et al. (2012) revealed 17 proteins with differential abundance of approximately two-fold or greater between schizophrenia and healthy subjects (listed in Table 2). These proteins involve diverse biological functions: oxidative stress, lipid metabolism, calcium-binding and other binding proteins, epidermal differentiation and integrity, protease inhibition, cell-cell adhesion, and glycolysis. The potential of sweat as a source for new schizophrenia biomarkers was highlighted, since only 5 proteins [albumin (ALB), alpha-2-glycoprotein 1 (AZGP1), clusterin isoform 1 (CLU), apolipoprotein D precursor (APOD), and gelsolin isoform b (GSN)] were found in common between sweat and serum.

Saliva allows non-invasive specimen collection, and it can sometimes be a good substitute for plasma or blood. Until now, only one study has investigated saliva as a potential source for schizophrenia and bipolar disease biomarkers using top down proteomics platform (RP-HPLC–ESI-MS; Iavarone et al., 2014). All significantly altered proteins and peptides were involved in innate immunity (α-defensins 1–4, S100A12, cystatin A and S-derivatives of cystatin B). However, as increased levels of these proteins were also found in association with neoplasms and infectious diseases, the authors concluded that this set of proteins has low diagnostic potential for schizophrenia.

One small study (Herberth et al., 2011) used LC-MS^E^ to investigate the proteomes of PBMCs, at baseline and after 72 h of stimulation by staphylococcal enterotoxin and CD28. Cells from antipsychotic-naïve subjects in the first episode of schizophrenia, compared with cells from healthy subjects, showed altered levels of some glycolytic enzymes at rest, and more after stimulation, while these alterations were not seen in PBMC from individuals with chronic schizophrenia and antipsychotic treatment. Curiously, the post-stimulation levels of ALDOC were greater in the cells from anti-psychotic-naïve schizophrenia subjects than in the cells from healthy subjects, while there was no reported abnormality of ALDOA, which in PBMC is an order of magnitude more abundant than ALDOC, and two orders of magnitude more abundant than ALDOB (see: http://moped.proteinspire.org/; Kim et al., 2014; Montague et al., 2015). As discussed elsewhere in this review, in schizophrenia, ALDOC is the most frequently abnormally-expressed protein identified in proteomic studies of the brain, where its expression is predominantly astrocytic, but some studies of schizophrenia find elevated levels and others find depressed levels (see below and Tables 1, **5**). An abnormal response of the C isoform in a cell that normally expresses predominantly the A isoform could point to a specific dysregulation of the C isoform in schizophrenia, but it could also indicate a general lability of the C isoform in the presence of independent abnormalities of glycolysis.

## The specificity of the candidate biomarkers for schizophrenia

To evaluate the overlap of the schizophrenia-associated candidate biomarkers with candidate biomarkers for other neurological diseases, we reviewed proteomic studies and reviews of several diseases such as bipolar disorder, major depressive disorder, Alzheimer's disease and Parkinson's disease.

We found 5 proteomics studies where both schizophrenia and bipolar disorder were compared with a non-psychiatric group (Novikova et al., 2006; Pennington et al., 2008; Behan et al., 2009; English et al., 2009; Föcking et al., 2011). Pennington et al. (2008) found 6 out of 66 proteins to be altered in both diseases, with cytoskeleton and synaptic-associated function (two forms of SEPT5, SEPT11, DPYSL3, TCP1), metabolism and CNS development (PHGDH). However, half of the proteins with altered abundance in bipolar disorder were cytoskeletal, metabolic or mitochondrial-associated proteins and some, such as TUBA, TUBB, NEFM, ATP5B, ENO2, PRDX2, PRDX6, ATP6V1A have been frequently found in schizophrenia studies (Table 1). Behan et al. (2009) identified three proteins involved in synapse-associated functions (LSAMP, BASP1, and STXBP1) while in a subsequent study from the same group (English et al., 2009), some intermediate filaments (NEFH, vimentin) and cytoskeleton-associated proteins (DPYSL2, DPYSL3, YWHAE) showed the same change in abundance in bipolar disorder as in schizophrenia. Novikova et al. (2006) found 5 proteins that were altered in both schizophrenia and bipolar disorder (CEBPZ, DECR2, BYSL, ANKARD, and ALDOC), associated with cell signaling, lipid and glucose metabolism, and other intracellular processes. The comparative proteomic analysis of hippocampus reported similar protein changes in schizophrenia and bipolar disorder with two-thirds of the proteins with altered abundances in schizophrenia showing the same trend in bipolar disorder as well (Föcking et al., 2011). These proteins were mostly involved in cytoskeletal and metabolic cellular mechanisms.

Two proteomic studies included major depressive disorder (MDD) in addition to schizophrenia and bipolar disorder. In one of the first proteomics studies of the brain, abundance of 3 proteins (DPYSL2, ALDOC, and GFAP) were altered in the frontal cortex in all three disorders (Johnston-Wilson et al., 2000). Altered levels of these proteins, associated with synaptic function, glycolysis, and cytoskeletal structure, respectively, were subsequently confirmed by most other proteomic studies of the brain in schizophrenia. A study of the protein changes in ACC in schizophrenia, MDD and bipolar disorder found that cytoskeletal and mitochondrial proteins are the most prominently altered in all of these disorders, but only 2 identified proteins (SRI, ALDOC) were found with changed levels in both schizophrenia and MDD (Beasley et al., 2006). The rest of the MDD - focused studies identified candidate biomarkers that to some extent overlap with those for schizophrenia (Martins-de-Souza, 2014). The biological functions implicated in MDD, such as energy metabolism, cellular transport, and cell communication and signaling are also the main pathways implicated in schizophrenia. However, several important differences that help in differentiation of these diseases on a protein level were observed: The disorders were associated with different pathways of energy metabolism (glycolysis is the main affected pathway in schizophrenia, while oxidative phosphorylation is more affected in MDD) and opposite changes of the same proteins were identified in both diseases (Martins-de-Souza et al., 2012a).

From the 93 proteins that have shown quantitative changes or modifications in 43 2-D based proteomic studies in 13 different brain regions in Alzheimer's disease between 1999 and 2010 (Korolainen et al., 2010), 34 proteins have been reported in schizophrenia brain proteomics studies as well. These 34 proteins point to the same disturbances in two major pathways: One is energy metabolism, especially glycolysis (ALDOA/C, ENO1/2, GAPDH, LDHB, PGAM1, PKM2, TPI1), Krebs cycle (ACO2, MDH1), mitochondrial function (ATP5A1, ATP5B), and oxidative stress (Piredoxins [PRDX2, 3, 6], SOD, CKB). The other pathway is cytoskeletal, through tubulins (TUBA1B, TUBA1C, TUBB), intermediate filaments (GFAP, NEFL,) cytoskeleton associated proteins (DPYSL2, YWHAE, YWHAG, STMN1,) and heat shock proteins (HSPA9, HSPA5, HSPD1, HSPA1A, HSPA2, HSPA8). Although the comparative proteomics studies of post-mortem human brains from Parkinson's disease are quite limited, the reported proteins showed some overlap with schizophrenia in neurofilaments (NEFL, NEFM), peroxiredoxins, and proteins involved in ATP synthesis (Srivastava et al., 2010).

The majority of the blood-based candidate proteins for schizophrenia are also involved in Parkinson's disease, Alzheimer's disease, and MDD. Several acute phase proteins (A2M, C3, SERPINA1, and ALB), proposed as candidate biomarkers for schizophrenia, were also found to be associated with Parkinson's disease, Alzheimer's disease, and MDD (Chiam et al., 2015). In addition, APOE and APCS were associated with both Parkinson's disease and Alzheimer's disease.

From the proposed candidate cerebrospinal fluid biomarkers for schizophrenia (Table 2), APOA1, APOA4, APOE, and PTGDS have been also proposed as candidate biomarkers for Alzheimer's disease and TTR (synthesized in the brain uniquely by choroid plexus, Herbert et al., 1986) as a candidate biomarker for Parkinson's and Alzheimer's diseases (Korolainen et al., 2010; Kroksveen et al., 2011).

## Cell-specific proteins

Since different cells produce different proteins, one could look to proteomics for evidence of specific cell types affected in schizophrenia. We therefore compared the proteins in Table 1 with lists of genes identified in a large study of transcriptional signatures of cell type in mouse CNS (Cahoy et al., 2008) and a comparison of microglia with circulating monocytes (Yamasaki et al., 2014). From the former report, we extracted 240 genes, of which 106 were preferentially expressed in astrocytes, 83 in oligodendrocytes, 43 in neurons, 5 in endothelial cells, and 3 in microglia. From the latter, we extracted 76 genes, of which 48 were preferentially expressed in microglia and 28 in monocytes. Of the 221 proteins reported as differentially expressed in schizophrenia (Table 1), mRNA sequences for 22 were also reported as characteristic of individual cell types (Table 3). These include the 2 proteins most frequently reported altered in schizophrenia, ALDOC (11 reports) and GFAP (9 reports), both expressed predominantly in astrocytes.

**Table 3:** **Proteins with differential abundance in schizophrenia reported as characteristic of individual cell types by transcriptional studies of mouse brain cells and mononuclear cells**.

**Gene**	**Cell type**	Descriptive Group^*^	Citations^**^										
ALDOC	astrocytes	C: 13/40; C: well-described; C: astrocyte-enriched	Martins-de-Souza et al., 2009d	Novikova et al., 2006	Johnston-Wilson et al., 2000	English et al., 2009	Martins-de-Souza et al., 2009a	Prabakaran et al., 2004	Smalla et al., 2008	Clark et al., 2006	Clark et al., 2007	Martins-de-Souza et al., 2009b	Martins-De-Souza et al., 2010c
GFAP	astrocytes	C: 13/40; C: well-described; C: astrocyte-enriched	Martins-de-Souza et al., 2009d	Martins-de-Souza et al., 2010a	Johnston-Wilson et al., 2000	Martins-de-Souza et al., 2009a	Pennington et al., 2008	Clark et al., 2006	Sivagnanasundaram et al., 2007	Martins-de-Souza et al., 2009b	Föcking et al., 2011		
HIST1H3G	astrocytes	C: developmental	Martins-de-Souza et al., 2009d										
HSPA1B	astrocytes	C: developmental	Nesvaderani et al., 2009										
PDE8B	astrocytes	C: developmental	English et al., 2009										
PYGM	astrocytes	C: developmental	Saia-Cereda et al., 2015										
SLC9A3R1	astrocytes	C: astrocyte-enriched	Martins-de-Souza et al., 2009d										
GNAQ	microglia	Y: MG-enriched	Smalla et al., 2008										
MAPK3	microglia	Y: MG-enriched	Martins-de-Souza et al., 2010b										
GAPDH	monocytes	Y: monocyte-enriched	Martins-de-Souza et al., 2009c	Martins-de-Souza et al., 2010a	Saia-Cereda et al., 2015	Martins-de-Souza et al., 2009b							
PGK1	monocytes	Y: monocyte-enriched	Martins-de-Souza et al., 2009a										
NEFL	neurons	C: well-described; C: neuron-enriched	Martins-de-Souza et al., 2009d	English et al., 2009	Martins-de-Souza et al., 2009a	Pennington et al., 2008	Clark et al., 2007	Saia-Cereda et al., 2015	Sivagnanasundaram et al., 2007	Föcking et al., 2011			
NEFM	neurons	C: neuron-enriched	Martins-de-Souza et al., 2009d	Martins-de-Souza et al., 2010a	English et al., 2009	Saia-Cereda et al., 2015	Sivagnanasundaram et al., 2007	Nesvaderani et al., 2009					
CAMK2B	neurons	C: neuron-enriched	Martins-de-Souza et al., 2010b										
GDA	neurons	C: 29/40; C: neuron-enriched	Pennington et al., 2008										
CNP	oligodendrocytes	C: oligodendrocyte-enriched	Martins-de-Souza et al., 2009c	Martins-de-Souza et al., 2009d	Prabakaran et al., 2004	Saia-Cereda et al., 2015							
MBP	oligodendrocytes	C: 27/40; C: well-described; C: oligodendrocyte-enriched C:developmental	Martins-de-Souza et al., 2009d	Martins-de-Souza et al., 2010a	Martins-de-Souza et al., 2009a	Saia-Cereda et al., 2015							
MOG	oligodendrocytes	C: 2/40; C: well-described; C: oligodendrocyte-enriched; C: developmental	Martins-de-Souza et al., 2009c	Martins-de-Souza et al., 2009d	Martins-de-Souza et al., 2010a	Saia-Cereda et al., 2015							
GSN	oligodendrocytes	C: 10/40; C: oligodendrocyte-enriched	Prabakaran et al., 2004	Saia-Cereda et al., 2015	Nesvaderani et al., 2009								
HAPLN2	oligodendrocytes	C: oligodendrocyte-enriched; C:developmental	Saia-Cereda et al., 2015										
MYO1D	oligodendrocytes	C: oligodendrocyte-enriched; C:developmental	Martins-de-Souza et al., 2009c										
PLP1	oligodendrocytes	C: 17/40; C: oligodendrocyte-enriched; C:developmental	English et al., 2009										

* *Descriptive Group refers to the heading of the table(s) from Cahoy et al. (2008) (C) or Yamasaki et al. (2014) (Y) in which the gene was listed*.

*[Number]/40 refers to the rank on the list of “Top 40” genes for that cell type*.

** *Citations are the references for proteomic studies reporting that the protein is altered in schizophrenia*.

**Figure 1:**
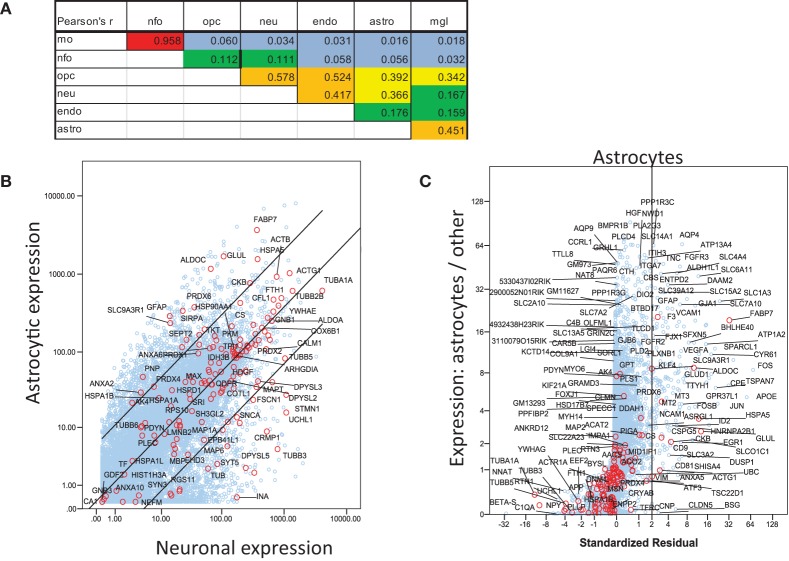
**Analysis of mouse RNAseq data from http://jiaqianwulab.org/resource.htm (Zhang et al., 2014; Dong et al., 2015). (A)** Correlation coefficients between cell types for all genes. **(B)** Linear regression of gene expression in ASTRO and NEU. Genes for proteins included in Table 1 are represented by red circles and labeled where space permits. Other genes are represented by blue circles. Center line represents best fit and outer lines 95% confidence limits after log transformation of data. **(C)** Multiple regression of ASTRO against other cell types. Linear regression of untransformed expression data for ASTRO against data for MO, OPC, NEU, and ENDO, was performed, and standardized residuals were computed. NFO were omitted from all multiple correlations because of their very high correlation with MO. Standardized residuals are potted on X-axis. The Y axis represents the ratio between expression level in ASTRO and the average expression levels in the other five cell types. Genes with standardized residual values above two (i.e., to the right of the vertical line) were considered enriched in astrocytes. Colors are as in **(B)**; both red and blue circles are labeled where space permits.

**Figure 2:**
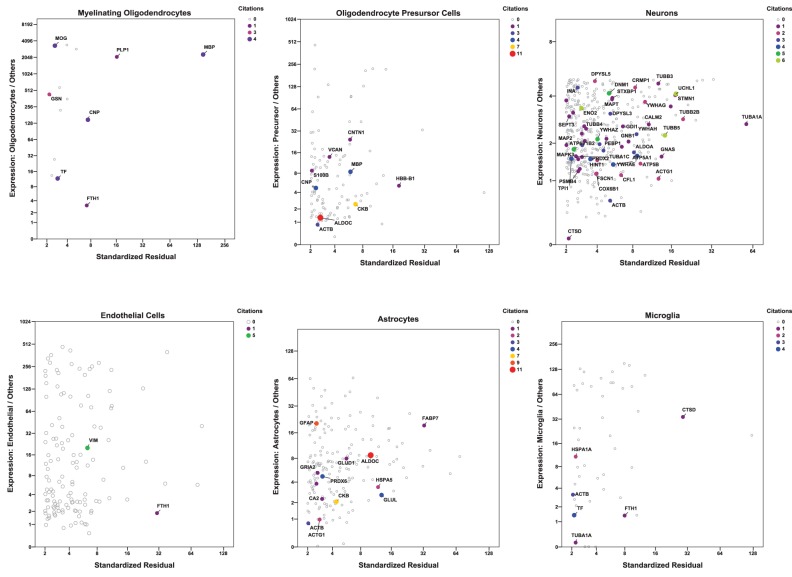
**Multiple regression was performed and plotted as in Figure 1C, except that only data points with standardized residuals >2 are shown**. The color of the symbols represents the number of proteomic studies in which the level of each protein was found differentially expressed in brain tissue from individuals with schizophrenia (Table 1). Only genes for proteins cited at least once are labeled. Because of their biological relationship, MO expression was not included as an independent variable in the regression of OPC, and OPC was not included in the regression of MO.

For a more inclusive analysis, we made use of a published dataset (http://jiaqianwulab.org/resource.htm) from mouse RNA sequencing (RNAseq) expriments (Zhang et al., 2014; Dong et al., 2015). This dataset includes expression levels of 22,462 genes from neurons (NEU), astrocytes (ASTRO), endothelial cells (ENDO), microglia (MGL), myelinating oligodendrocytes (MO), newly formed oligodendrocytes (NFO), and oligodendrocyte precursor cells (OPC). Expression levels in different cell types were highly correlated (Figure 1A), so gene expression in two cell types can be compared, and genes enriched in either cell type, relative to the other, can be determined by linear regression (e.g., Figure 1B). Similarly, one cell type can be compared with several others simultaneously by performing multiple linear regression and identifying the genes with standardized residuals greater than 2 (Figure 1C). By regression of each of 6 brain cell types against the others we found 714 such genes, of which 78 code for proteins that proteomic studies have identified as affected in schizophrenia. These include 7 of 14 genes expressed preferentially in MO, 13/174 in ASTRO, 9/108 in OPC, 54/362 in NEU, 2/129 in ENDO, and 6/46 in MGL (Figure 2). These regressions are restricted to comparisons amongst different types of brain cells; they do not imply any comparison with expression of transcripts in other organs.

The comparisons among cell types are based on RNA, which does not necessarily correlate with levels of the encoded protein. Furthermore, they are based on cells from mice, not humans. However, the specificities (at least within the brain) of the most commonly cited astrocytic proteins, ALDOC and GFAP are well-established by immunohistochemistry on the human brain [except for the presence of ALDOC in cerebellar Purkinje cells (Thompson et al., 1982; Kumanishi et al., 1985), which were not included in any of the proteomic studies]. Similarly, the specificities of the most commonly cited oligodendroglial proteins, MBP, CNP, MOG, and GSN (Tanaka and Sobue, 1994), all associated with myelin, are also well-established by immunohistochemistry.

## Conclusion and future perspectives in schizophrenia proteomics research

We have attempted to review all studies of schizophrenia by proteomics, including studies of autopsy brain tissues and bodily fluids. The data lead us to conclude that there are important issues regarding the proteomics platforms and samples that make comparisons across studies quite difficult, especially when different platforms are used. More comparisons with other psychiatric illnesses are needed to address specificity. Proteomic studies have been confined to a small number of brain regions, with little attention to white matter.

Overall, however, the data from brain tissue point to prominent changes in metabolic pathways and cytoskeleton. The majority of studies of bodily fluids point toward immune response, lipid metabolism and to a lesser extent, dysregulation of glucose metabolic pathways. The discrepancy between findings in tissue and body fluids has been observed generally in biomarker studies, particularly in studies of cancer (Hanash et al., 2008). This lack of tissue biomarker detection in the circulation may be caused by low levels of release of disease-associated proteins from tissues to body fluids, where they are susceptible to masking by highly abundant blood proteins. Alternatively, circulating proteins associated with a particular disease may be too dilute for detection by the methods used for proteomics.

The comparison of the candidate biomarkers for schizophrenia with candidate biomarkers for other neurological diseases, such as bipolar disorder, MDD, Alzheimer's disease, and Parkinson's disease reveal overlap. This could be due to common disturbances in energy pathways and cytoskeletal function, or to bias associated with the proteomics method used. Most of the proteomics studies of brain tissues reviewed here and most of the proteomics studies of common neurological disorders, reviewed extensively elsewhere (Korolainen et al., 2010; Srivastava et al., 2010), have been based on2-D gel-based platforms. This approach is known to resolve only a limited collection of highly abundant and soluble proteins (Petrak et al., 2008; Wang et al., 2009). These proteins are mainly involved in central metabolism, protein production (e.g., ribosomal proteins, some RNA binding proteins, translation factors), protein conformational control and degradation (chaperones, disulfide isomerases, proline isomerases, proteasome subunits), cytoskeleton (including some cytoskeleton modifying proteins), adaptor proteins (14-3-3, annexins), and oxidative stress response (catalase, superoxide dismutases, peroxiredoxins, glutathione transferases). This limited scope of 2-D gels might explain why so many so called “specific” markers of various neurological diseases belong to the same classes. However, the research using shotgun label-free approaches, in comparative brain studies with MDD, provides a more detailed and specific overview of the energy metabolism pathways involved in the disease, with clues that these pathways are different from those implicated in schizophrenia (Martins-de-Souza et al., 2012a). Quantitative shotgun proteomics with data-independent acquisition (MS^E^) coupled with wave ion mobility has been applied to a wide variety of samples including cells, tissues, clinical samples, and affinity pull downs, with power and effectiveness firmly established and extensively validated by many independent groups (Brown, 2014). Therefore, future extensive studies using proteomics platforms with wider dynamic range, such as label-free data-independent (MS^E^) shotgun approach or MALDI MSI combined with high resolution top-down tandem MS identification could provide a more specific picture of the affected pathways in schizophrenia.

Assessment of the cell specificity of candidate biomarkers for schizophrenia did not point to exclusive dysfunction of one specific brain cell type. However, transcripts for 35% (78/221) of the proteins reported as affected in schizophrenia are enriched in one or more cell types in mouse brain. Since the vast majority (>95%) of mouse genes are not selectively expressed in any one type of brain cell compared with the others, there is clearly a preferential involvement of genes selectively expressed by specific types of brain cells. Among these 78 proteins, twice as many are highly expressed in neurons as in glia, but the glial proteins are found more consistently. Overall, ~50% of citations are to proteins not specifically expressed by individual types of brain cells, 30% to proteins preferentially expressed by neurons, and 20% to proteins predominantly expressed by glia. The most frequently referenced proteins are ALDOC (astrocytic), followed by GFAP (astrocytic) and DPYSL2 (nonspecific), followed by NEFL (nonspecific) and then CKB (oligodendroglial precursor and astrocytic). It is important to remember that the expression profiles on which this analysis relies are based solely on mRNA in mouse brain cells, since quantitative data on proteins in individual cell types from human brain are not available.

Studies of white matter, which is nearly devoid of neuronal cell bodies but abundantly, populated with oligodendroglia, oligodendroglial precursor (NG2) cells, astrocytes, and microglia showed some abnormalities of oligiodendroglial protein levels, with considerable variation across studies, and little or no replication of more consistent deficits in oligodendroglial mRNA. Abnormal content of astrocytic proteins in white matter was somewhat more consistent, with two studies reporting altered levels of ALDOC, and one reporting abnormal levels of GFAP. However, the picture changes somewhat when one looks beyond the handful of studies restricted to white matter. Gray matter includes myelin and all types of glia, and cortical samples cut macroscopically from frozen brain slices will almost inevitably include some white matter, intentionally or otherwise, since the cortex curves in 3 dimensions. The 7 studies reporting alterations in MBP, MOG, CNP, or GSN are listed in Table 4. In schizophrenia, levels of all 4 were depressed in 1 study of corpus callosum, while the 4 studies of neocortex found depressions of 1, 2, 2, and 3 of the proteins, respectively, with none elevated. Two studies, one of hippocampus and one of thalamus, reported elevation of GSN and MOG, respectively. Overall, these results corroborate the numerous reports from RNA and targeted protein studies of myelin-related deficits in schizophrenia. By contrast, while abnormalities of the astrocytic proteins ALDOC and GFAP are the most frequently reported, both are up in some studies and down in others, concordantly in some studies and discordantly in others (Table 5). There are many possible explanations, which must include the very varied functions of astrocytes in healthy and diseased brains.

**Table 4:** **Oligodendroglial proteins most commonly cited**.

**Region**	**MBP**	**CNP**	**MOG**	**GSN**	**References**
BA9 gray and white matter		↓		↓	Prabakaran et al., 2004
BA46		↓	↓		Martins-de-Souza et al., 2009c
Corpus callosum	↓	↓	↓	↓	Saia-Cereda et al., 2015
BA38, left	↓	↓	↓		Martins-de-Souza et al., 2009d
BA46, left	↓				Martins-de-Souza et al., 2009a
Hippocampus				↑	Nesvaderani et al., 2009
Mediodorsal thalamus			↑		Martins-de-Souza et al., 2010a

*Proteomics studies of brain reporting altered levels of myelin-associated proteins MBP, CNP, MOG, or GSN. Arrows as in Table 1*.

**Table 5:** **Astrocytic proteins most commonly cited**.

**Schizophrenia relative to nonpsychiatric**		**GFAP**			
		**Higher**	**Lower**	**Neither**	**Total**
ALDOC	Higher	1	1	2	4
	Lower	1	2	4	7
	Neither	2	2		4
	Total	4	5	6	15

*Numbers of studies from Table 1 reporting differences between schizophrenia and non-psychiatric cases in GFAP, ALDOC, or both. Since most reports do not include tabulations of all identified proteins, the category of “Neither” higher nor lower does not distinguish failure to identify the protein and a failure to find a significant difference between schizophrenia and non-psychiatric cases*.

At this time, exhaustive proteomic studies are new, and their interpretation is far from straightforward. This difficulty would be alleviated if researchers published entire datasets, rather than just positive findings. This is especially important for proteomics because, in contrast to mRNA expression studies, the total set of identified proteins varies dramatically between studies, so that proteins that are affected by disease in one study may not be detected at all in another. The potentially confounding influence of pre- and postmortem factors such as tissue pH, postmortem interval, medication exposure, and in particular, agonal state must always be reported and considered. Detailed and complete clinical and demographic data should also be included. Discovery proteomic studies, using well-matched sub-pools of disease and control subjects, should be followed by validation of important findings with other methods *in larger samples*, not just in those employed for discovery. Overall, the proteomics studies of schizophrenia so far have paved the way for future research that should contribute to greater understanding of the molecular mechanisms of the disease and identify biomarkers for clear and effective diagnosis, nosology, prognosis, and therapy. This is possible by using well-defined samples, larger cohorts, combinations of proteomics techniques to overcome limitations associated with individual techniques, and extensive validation.

## Author contributions

KD collected literature, assembled Tables 1 and 2, wrote the first draft of the manuscript, and edited subsequent drafts. IM collected literature, assembled Tables 1 and 2, and edited the manuscript. AJD outlined the project, compared the human and mouse data, prepared the figures and Tables 3–5, and revised and edited the manuscript.

## Funding

This study was supported by NIH Research Grants R01 MH098786, funded by the Fogarty International Center and the National Institute of Mental Health, and R01 MH064168, funded by the National Institute of Mental Health.

### Conflict of interest statement

The authors declare that the research was conducted in the absence of any commercial or financial relationships that could be construed as a potential conflict of interest.
